# Zirconium Ions Up-Regulate the BMP/SMAD Signaling Pathway and Promote the Proliferation and Differentiation of Human Osteoblasts

**DOI:** 10.1371/journal.pone.0113426

**Published:** 2015-01-20

**Authors:** Yongjuan Chen, Seyed-Iman Roohani-Esfahani, ZuFu Lu, Hala Zreiqat, Colin R. Dunstan

**Affiliations:** Biomaterials and Tissue Engineering Research Unit, School of AMME, The University of Sydney, Sydney, New South Wales 2006, Australia; Inserm U606 and University Paris Diderot, FRANCE

## Abstract

Zirconium (Zr) is an element commonly used in dental and orthopedic implants either as zirconia (ZrO_2_) or in metal alloys. It can also be incorporated into calcium silicate-based ceramics. However, the effects of *in vitro* culture of human osteoblasts (HOBs) with soluble ionic forms of Zr have not been determined. In this study, primary culture of human osteoblasts was conducted in the presence of medium containing either ZrCl_4_ or Zirconium (IV) oxynitrate (ZrO(NO_3_)_2_) at concentrations of 0, 5, 50 and 500 µM, and osteoblast proliferation, differentiation and calcium deposition were assessed. Incubation of human osteoblast cultures with Zr ions increased the proliferation of human osteoblasts and also gene expression of genetic markers of osteoblast differentiation. In 21 and 28 day cultures, Zr ions at concentrations of 50 and 500 µM increased the deposition of calcium phosphate. In addition, the gene expression of BMP2 and BMP receptors was increased in response to culture with Zr ions and this was associated with increased phosphorylation of SMAD1/5. Moreover, Noggin suppressed osteogenic gene expression in HOBs co-treated with Zr ions. In conclusion, Zr ions appear able to induce both the proliferation and the differentiation of primary human osteoblasts. This is associated with up-regulation of BMP2 expression and activation of BMP signaling suggesting this action is, at least in part, mediated by BMP signaling.

## Introduction

Zirconium (Zr) is one of the more common trace elements present in the environment [1]. It is a metallic element with a valency of 4 that is normally present in human bone and tissues at a trace level in the range 2–20 mg/kg body weight with an estimated average daily intake in humans of 3.5 mg [1]. Toxicity of Zr has been assessed as low to moderate in animals [1].

Zr containing materials, predominantly as the stable and biologically inert zirconium oxide (ZrO_2_) and metal alloys, have been widely used in dental applications and as coatings for orthopaedic implants due to their contributions to biocompatibility, increased mechanical strength, and high corrosion resistance [2–5].

Recently we have developed a calcium silicate-based ceramic, baghdadite (Ca_3_Zr[O_2_Si_2_O_7_]) (Patent Application # 792007905843) incorporating Zr which can be released as Zr ions into aqueous media. This ceramic was more labile than the ZrO_2_ and has been shown to release Zr into solutions simulating body fluids at concentrations in the range 10–100 µM [6]. This ceramic when fabricated as a porous scaffold, has been shown to have excellent osteogenic bio-activity in vitro and in vivo [6, 7] when compared to calcium silicate ceramics. This ceramic material has been tested in a critical sized bone defect model in the rabbit and appears to be superior in promoting osteogenesis than the currently used clinical implant materials containing calcium triphosphate and hydroxyapatite [6]. We further demonstrated that Baghdadite scaffolds can modulate the crosstalk between adipose stem cells (ASCs) and primary human osteoblasts (HOBs) to promote osteogenic gene expression in both ASCs and HOBs in an indirect co-culture system [8].

However the mechanism for this enhanced bioactivity has not been identified, and, in particular, the in vitro effects of Zr ions on human osteoblasts have not previously been studied. Review of the literature indicates an absence of studies evaluating the effects of Zr ions in cells of the osteoblast lineage. There has been one limited study reported in the literature examining the in vitro toxicity of Zr on the osteoblast-like cell line MG63 which showed cell toxicity in the millimolar concentration range but which did not investigate the effects of Zr ions on the proliferation and differentiation of these cells at lower more clinically relevant concentrations [9]. The response of osteoblast-like cell lines and human osteoblasts have been assessed when grown on Zr containing alloys and ceramics [7, 10–18] but in none of these is it possible to differentiate surface morphology effects and the influence of other material components from those of Zr itself.

Various trace elements have been found to have activity on bone cells. Strontium [19] and fluoride ions [20] have each been shown to have osteogenic properties for in vitro osteoblast cell cultures and when administered systemically, and gallium has been found to inhibit the activity of osteoclasts and inhibit bone resorption [21]. Zr ions have not been assessed for osteogenic properties and it is thus possible that the benefits of incorporating Zr in implanted materials could include local direct effects on bone formation. Two forms of Zr that are readily soluble in aqueous solutions are Zirconium (IV) oxynitrate (ZrO(NO_3_)_2_) and zirconium chloride (ZrCl_4_) and these are used in the present study to generate culture media containing Zr ions. The determination of the actual ionic species in aqueous solutions is complex as simple Zr^4+^ ions are absent in aqueous solutions where extensive hydrolysis leads to the formation of oligomeric species such as Zr_4_(OH)_4_^8+^(aq) [22] but for the purposes of this current study, further characterization of the actual ionic species present will not be addressed.

In this study we show that Zr ions do have the ability to promote the proliferation and differentiation of human osteoblasts in vitro and that this effect is associated with, and may be mediated by, up-regulation of BMP2 expression and increased BMP signaling.

## Materials and Methods

### Isolation and culture of primary HOBs

The use of primary human osteoblasts isolated from unneeded surgically removed bone was approved by the Human Ethics Committee of the University of Sydney. As the source of the bone was from minors, written informed consent was obtained from parents/guardians of the subjects. Both the protocol and the consent procedures were approved by the Human Ethics Committee of the University of Sydney. HOBs were isolated previously described [23] from discarded human vertebral trabecular bone from young (<15 years) healthy adolescents undergoing operations correcting scoliosis. Briefly, bone was cut into 1 mm^3^ pieces and washed with phosphate-buffered saline (PBS). Bone pieces were digested in 0.02% trypsin (Sigma-Aldrich) for 90 minutes at 37°C. The digested cells were cultured in complete α-minimal essential medium (αMEM, Gibco Laboratories) supplemented with 10% (v/v) heat-inactivated fetal calf serum (FCS, Gibco Laboratories), 100 units/ml penicillin and streptomycin (Gibco Laboratories) and 1 mM L-ascorbic acid phosphate magnesium salt (Wako Pure Chemicals) at 37°C with 5% CO_2_ in a humidity atmosphere. Only Passage 2 or 3 HOB cells were used in this study.

### Chemicals and HOB treatments

ZrO(NO_3_)_2_, ZrCl_4_ and sodium nitrate (NaNO_3_) were purchased from Sigma-Aldrich. Zr compound solutions were made by dissolving the chemical powders in PBS (pH 7.4) and sterilized by filtration through 0.2 µm pore syringe filters (Millipore). The solutions of ZrO(NO_3_)_2_ and ZrCl_4_ were stored at 4°C as stocks at concentrations of 5, 50 and 500 mM respectively. For use in treatment of HOB cell cultures, the stock solutions were diluted to final concentrations of Zr in the medium of 5, 50 and 500 µM. HOBs were incubated in the complete medium with addition of an appropriate volume of PBS alone for untreated control conditions (refer as “Control” in the text and figures below). An additional control containing 50 µM concentration of NaNO_3_ in PBS was used in these studies to determine whether the NO_3_- ions in the ZrO(NO_3_)_2_ containing culture media may have impacted HOB osteogenesis. The medium with the treatments was refreshed every 3 days.

### Methylthiazolyldiphenyl-tetrazolium bromide (MTT) assay

HOB cells at Passage 3 were seeded in 96-well plates, 1x10^4^ cells/well. Cells were incubated in the complete αMEM containing Zr chemicals ZrO(NO_3_)_2_ and ZrCl_4_ at different concentrations with an untreated control and a NaNO_3_-treated control for 1, 3 and 7 days. In each treatment group, a MTT (Sigma-Aldrich) assay was used to evaluate the number of viable cells present. HOBs were incubated with 2.5 mg/ml MTT for 2 hours at 37°C. The MTT solution was then removed and replaced with 100 µl DMSO to solubilize the formazan dye formed. After the plate was shaken on the shaker for 10 minutes, the absorbance of each well was read at 570 nm in a microplate photometer (Thermo Multiscan EX).

### Mineralized matrix formation

HOBs were seeded in 24-well plates at the density of 5x10^4^ cells/well and cultured in the complete αMEM medium. Cells were treated with Zr chemicals, complete medium as an untreated control, or a solution containing NaNO_3_ for 21 and 28 days. Medium with the treatment was refreshed every 3 days. Alizarin Red S Staining was carried out as previously described [24]. In brief, at each time point, cells were washed with Millipore water and incubated in Alizarin Red S Solution (pH 4.3) for 30 minutes at room temperature. Then cells were washed in water for four times and stored in water for image acquisition using a bright field light microscope (Olympus).

### Quantitative real time polymerase chain reaction

Total RNA extraction was carried out as previously described [25]. Briefly, HOBs were treated with and without Zr chemicals; or HOBs were co-incubated with Noggin (500 ng/ml, Sigma-Aldrich) and ZrCl_4_ at the concentrations of 50 and 500 µM, for 3 and 7 days. The medium was removed and cells were washed with ice-cold PBS. Trizol Reagent (Sigma-Aldrich) was added into each well. The following steps were performed after precipitation of the RNA in isopropanol and washing of the RNA pellets in 70% ethanol. RNA was dissolved in DEPC-H_2_O and the concentration of RNA was quantified on a NanoDrop spectrophotometer (Thermo Fisher). First-strand cDNA was synthesized from 1µg of RNA using a Tero cDNA synthesis kit (Bioline) according to the manufacturer’s instructions. Osteogenic genes, including osteopontin (OPN), bone sialoprotein (BSP), runt-related transcription factor 2 (Runx2), osteocalcin (OC), alkaline phosphatase (ALP), bone morphogenetic protein 2 (BMP2), BMP receptor 1a (BMPR1a), BMPR1b and BMPR2 were analyzed using a Rotor-gene 6000 (Corbett Life Science). Relative gene expression was normalized with the house-keeping gene 18S. Primers for the genes are listed in Table 1.

**Table 1:** Primers Used for Real-Time Polymerase Chain Reaction.

**Gene**	**Sequence (5’–3’)**	**Melting Temperature (°C)**
**18S**	F GTAACCCGTTGAACCCCATT	60
	R CCATCCAATCGGTAGTAGCG	
**Osteopontin**	F TTCCAAGTAAGTCCAACGAAAG	60
	R GTGACCAGTTCATCAGATTCAT	
**BSP**	F ATGGCCTGTGCTTTCTCAATG	60
	R GGATAAAAGTAGGCATGCTTG	
**Runx2**	F ATGCTTCATTCGCCTCAC	60
	R ACTGCTTGCAGCCTTAAAT	
**Osteocalcin**	F ATGAGAGCCCTCACACTCCTCG	60
	R GTCAGCCAACTCGTCACAGTCC	
**ALP**	F CGTGGCTAAGAATGTCATCATGTT	60
	R AGGGGAACTTGTCCATCTCC	
**BMP2**	F AGTTGCGGCTGCTCAGCATGTT	60
	R CCGGGTTGTTTTCCCACT	
**BMPR1a**	F TTTATGGCACCCAAGGAAAG	60
	R TGGTATTCAAGGGCACATCA	
**BMPR1b**	F AAAGGTCGCTATGGGGAAGT	60
	R GCAGCAATGAAACCCAAAAT	
**BMPR2**	F CATCCGAACCCTCTCTTGAT	60
	R TGCATAAAGATCCATTGGGA	

BSP, bone sialoprotein; Runx2, runt-related transcription factor 2; ALP, alkaline phosphatase; BMP2, bone morphogenetic protein 2; BMPR, BMP receptor

### Protein extraction and Western Blot analysis

HOB cells were seeded on 6-well plates at a density of 5x10^4^ cells/well and cultured with Zr solutions at the final concentrations of 5, 50 and 500 µM for 7 days. Untreated controls and NaNO_3_-treated controls were used. At D7, cells were washed with ice-cold PBS and lysed in Radio Immuno Precipitation Assay (RIPA) lysis buffer containing 20 mM Tris-HCl (pH 7.5), 1 mM ethylenediaminetetraacetic acid, 1 mM ethylene glycol tetraacetic acid (EGTA), 150 mM NaCl, 1% Triton X-100, 1% protein inhibitor cocktail solution (PICS, Sigma-Aldrich) and phosphatase inhibitor (Roche). Protein concentrations were measured using the bicinchoninic acid (BCA) assay kit (Pierce). 10 µg protein samples in 4x sample buffer (WesternBreeze, Invitrogen) were heated at 70°C for 10 minutes and separated on 8%–12% sodium dodecylsulphate (SDS)-polyacrylamide electrophoresis gels (WesternBreeze, Invitrogen). Proteins were transferred to the polyvinylidene fluoride (PVDF) membrane and washed with TBS-T (20 mM Tris-HCl, pH 7.6 and 137 mM NaCl) containing 0.1% Tween 20. After blocking in TBS-T with 1% bovine serum albumin (BSA) for one hour at room temperature, the membrane was incubated with the primary antibodies, anti-BMP2 (Abcam, 1:1000), anti-SMAD1(Cell signaling, 1:1000) or anti-phospho-SMAD1/5 (p-SMAD1/5, Cell signaling, 1:500) in blocking buffer on the shaker at 4°C overnight. Following three washes, the membrane was incubated in the secondary antibody conjugated with Alkaline Phosphatase (AP) (WesternBreeze, Invitrogen) for one hour at room temperature. Chemilluminescent reagent (WesternBreeze, Invitrogen) was used to observe the protein bands under the Bio-Rad ChemiDoc MP Imaging System (Bio-Rad). For β-actin detection, the same membrane was stripped in the stripping buffer containing 60 mM Tris-HCl (pH6.8), 7% β-mercaptoethanol and 2% SDS at 50°C for 30 minutes under gentle agitation. The membrane was then incubated with anti-β-actin antibody (Abcam, 1:5000) for the loading control after blocking in the blocking buffer for one hour.

### Immunocytochemistry

HOBs were seeded on the coverslips and incubated with Zr solutions at 5, 50 and 500 µM, or NaNO3 solution at 50 µM or complete medium for 7 days. After washing in PBS for three times, cells were fixed in 4% paraformaldehyde for 20 minutes. Immunostaining of the treated HOBs was carried out using anti-Ki67 (1:100, Abcam), anti-BMP2 (1:500, Abcam), anti-p-SMAD1/5 (1:100, Cell Signaling) primary antibodies to detect protein activation through phosphorylation. The antibody, anti-p-SMAD1/5 (S463/S465), used recognizes phosphorylated forms of both SMAD1 and SMAD5 when phosphorylated at Ser463 and Ser465. The incubation with the primary antibody was processed in a humidity chamber overnight at 4°C. Ki67 reactivity was visualized with the secondary antibody anti-rabbit conjugated with horse radish peroxidase (HRP, Abcam) and 3,3’-diaminobenzidine (DAB) substrate. Hematoxylin was used for the nuclear counterstaining. The images were acquired under the bright field light microscope (Olympus). The immunofluorescence staining of BMP2 and p-SMAD1/5 was analyzed by incubating with the secondary antibody of anti-rabbit conjugated with Alexa-488 (Abcam) and then with DAPI for nucleus staining. The immunofluorescence was visualized under the fluorescent microscope (Olympus).

### Statistical analysis

Data from this study were obtained from three independent experiments and are represented as mean ± SD. Statistical analysis of data was performed by One-way ANOVA and Dunnett’s Post Hoc Test using a SPSS statistical software package (IBM Version 21).

## Results

### Zr ions increase HOB cell proliferation

**Figure 1:**
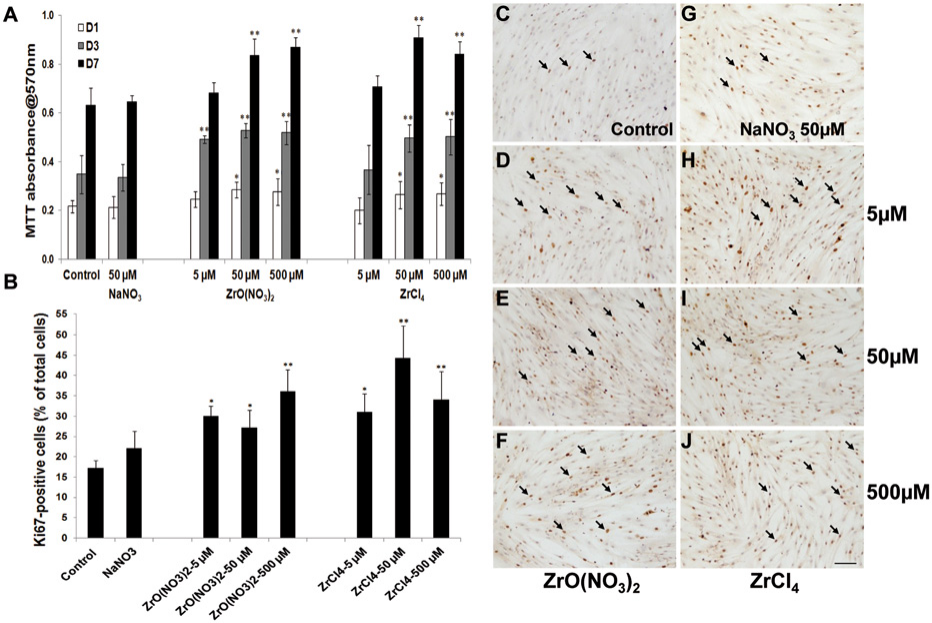
Zirconium ion promotes HOB proliferation. (A) The MTT assay was used to determine the HOB viability after HOBs were treated with zirconium solutions with different concentrations for 1, 3 and 7 days. Compared to the control and NaNO3-treated control, higher concentrations of zirconium significantly increased HOB viability. ZrO(NO_3_)_2_ at 5µM increased cell proliferation at D1 and D3 but not D7, whereas ZrCl_4_ at 5µM had no effects on HOB proliferation. However, higher concentrations of both of zirconium solutions significantly increased cell proliferation at D1, D3 and D7. Between the time points, viable cell number in each treatment group are significantly increased (p<0.01). (B) The graph shows the quantification of Ki67 positive cells to total cells. Compared to the control, the proportion of Ki67-positive cells is significantly increased in all treatment groups. There were no significant differences observed between the treatment groups at the different concentrations. (C-J) Representative images from each treatment group following KI67 immunostaining. Arrows indicate the positive staining of cells with the Ki67 antibody (C). Scale bar: 100 µm. Data are Mean±SD of three separate experiments. * p<0.05, **p<0.01 versus control.

The effects of Zr treatments on the number of viable HOBs were examined using a MTT assay. HOBs at Passage 3 were treated with ZrO(NO_3_)_2_ or ZrCl_4_ solutions at the final concentrations in the medium of 5, 50 and 500 µM and incubated for 1, 3 and 7 days, while the complete medium or NaNO_3_ solution at 50 µM in medium was used as an untreated control and a control for the nitrate content of ZrO(NO_3_)_2_. Compared to the untreated control and the NaNO_3_ control, at D1, D3 and D7, ZrO(NO_3_)_2_ and ZrCl_4_ at all concentrations greater than 5 µM significantly stimulated HOB proliferation. At D3 this increase was also significant for ZrO(NO_3_)_2_ at 5 µM (Fig. 1A). At D3 and D7 viable cell number increased progressively in each treatment and control group (p<0.01). There was no difference in the absorbance between the control group and NaNO_3_-50 µM group at D1, D3 and D7.

To further investigate the effects of Zr on HOB proliferation, immunostaining was carried out to assess the cell proliferation marker Ki67 labeling in HOBs. Increased Ki67 staining was observed in the nucleus in HOBs in Zr treatment groups (Fig. 1D-F, H-J) relative to the control groups (Fig. 1C,G). The quantification of Ki67-positive cells in total cells showed that, compared to the controls, Ki67-positive cells in ZrO(NO_3_)_2_ and ZrCl_4_-treated HOBs at D7 were significantly increased in all treatment groups (Fig. 1B), by 20–50%. There was no difference that was observed between the treatment groups at different concentrations. The Ki67 results confirm that the increase in viable cell number detected by MTT results, at least in part, from increased HOB cell proliferation.

### Zr ions induce mineralization in HOB cultures

**Figure 2:**
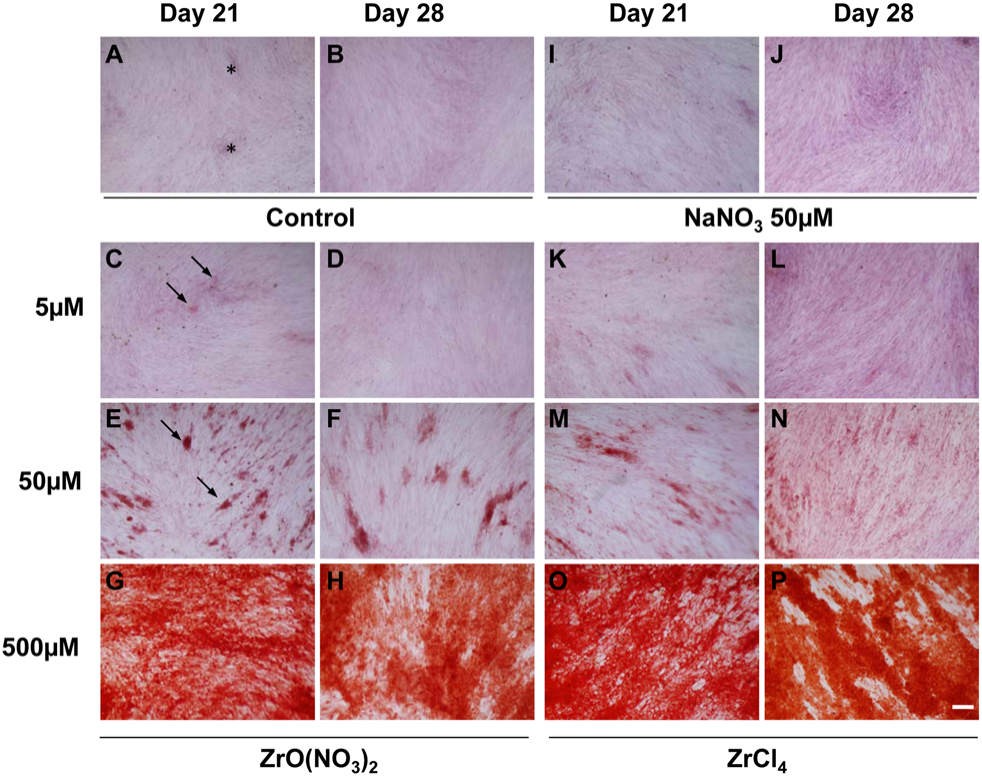
Effects of zirconium ion on mineralization of HOBs at D21 and D28. (A, B) In the controls, patchy weak Alizarin Red S staining (asterisks, A) was observed in HOBs at Day21 and D28. (C-H) ZrO(NO_3_)_2_ and (K-P) ZrCl_4_ dramatically promoted the formation of mineralized matrix (arrows, C, E), compared to the control (A, B) and NaNO_3_-treated control (I and J). Scale bar: 100 µm.

The induction of matrix mineralization in HOB cultures is consistent with the progress of differentiation to the formation of mature osteoblasts capable of bone matrix formation. To evaluate Zr ion effects in the HOB maturation, HOBs were incubated with Zr solutions and control media for 21 and 28 days with the medium changes every 3–4 days. Alizarin Red S Staining was used to analyze mineralized matrix formation in the treated HOB cultures. As shown in Fig. 2, the bone nodule formation in the HOB cells treated with Zr solutions was increased in a dose-dependent manner with effects seen at both Day 21 and Day 28. At Day 21 and Day 28, there were small patches of cells that were weakly stained with Alizarin Red (asterisks, Fig. 1A) appearing in the untreated control and NaNO_3_-treated control. Compared to the controls, the bone nodules formed in HOBs treated with ZrO(NO_3_)_2_ and ZrCl_4_ at 50 µM exhibited stronger staining (arrows, Fig. 1 E,F,M,N). The staining of HOBs were observed to be much stronger in the whole cell area and were greatest at the concentration of 500 µM (Fig. 1G,H,O,P) indicating Zr ions stimulated matrix mineralization by HOBs.

### Zr ions up-regulate osteoblast genetic markers and BMP2 gene expression in HOBs

As Zr was shown to increase HOB cell proliferation and matrix mineralization, we next analyzed specific genes associated with osteoblast differentiation in the Zr-treated HOB cells to validate the induction by Zr of differentiation into the osteoblast lineage. Total RNA was extracted from HOBs cultured in different Zr solutions or control media for 3 and 7 days, and real-time PCR was carried out to evaluate the expression of the osteoblast marker genes OPN, BSP, Runx2 and OC.

**Figure 3:**
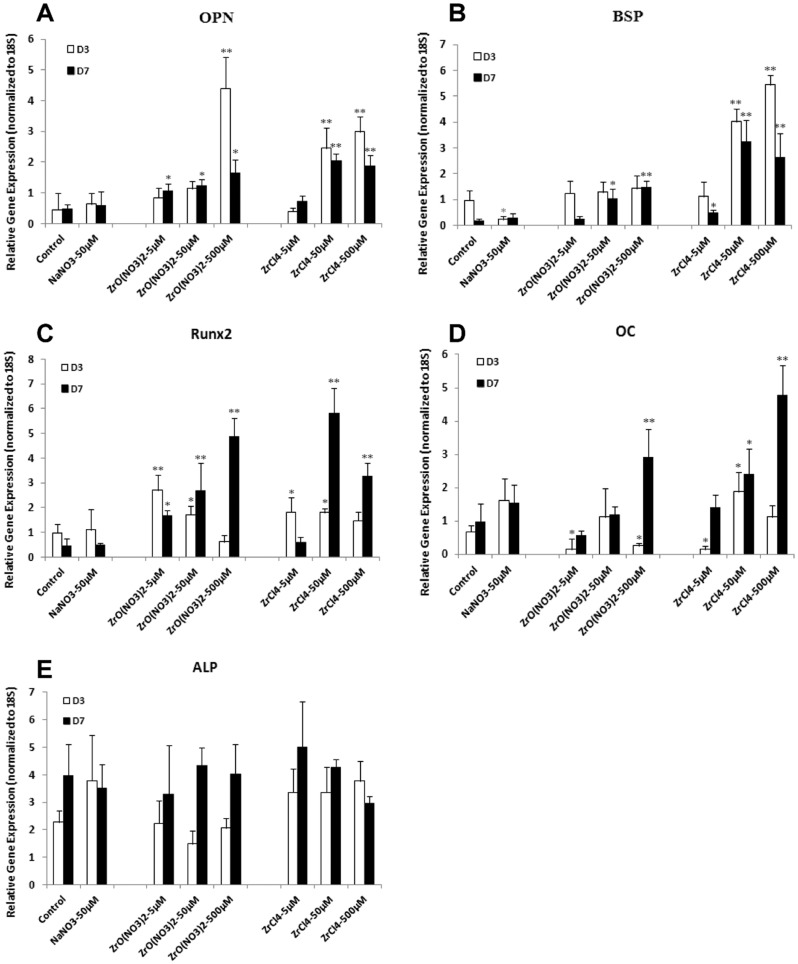
Zirconium ions up-regulate osteoblast genes in HOBs. Real-time PCR was used to analyze the expression of osteoblast genes in zirconium-treated HOBs. (A) At D7, OPN gene expression was significantly up-regulated in HOBs treated with ZrO(NO_3_)_2_ at concentrations of 5, 50 and 500 µM, compared to the controls. At D3, only ZrO(NO_3_)_2_ at 500 µM increased OPN expression. ZrCl_4_ at 50 and 500 µM increased OPN expression at D3 and D7, but not 5 µM. (B) Compared to the controls, ZrO(NO_3_)_2_ at 50 and 500 µM up-regulated BSP expression at D7, but not at D3; whereas ZrCl4 at 50 µM and 500 µM increased BSP expression at D3 and D7. (C) At D3, ZrO(NO_3_)_2_ and ZrCl_4_ at 5 and 50 µM increased Runx2 expression. At D7, at all three different concentrations, ZrO(NO_3_)_2_ increased Runx2 expression; However, ZrCl_4_ increased Runx2 expression only at 50 and 500 µM. (D) ZrO(NO_3_)_2_ and ZrCl_4_ at 500 µM increased OC expression at D7. At D3, OC expression was up-regulated by ZrCl_4_ at 50 µM; However it was suppressed by ZrO(NO_3_)_2_ at 5 and 500 µM and ZrCl_4_ at 5 µM. (E) ALP expression was up-regulated by ZrCl_4_ at 50 µM at D3 but no consistent evidence of regulation of ALP gene expression was seen. OPN, osteopontin; BSP, bone sialoprotein; OC, osteocalcin; ALP, alkaline phosphatase. *p<0.05, **p<0.01 vs control.

At D7, OPN gene expression was significantly up-regulated in HOBs treated with ZrO(NO_3_)_2_ at the three different concentrations of 5, 50 and 500 µM (Fig. 3A), compared to the controls. At D3, only ZrO(NO_3_)_2_ at 500 µM increased OPN expression. Similarly, ZrCl_4_ at 50 and 500 µM increased OPN expression at D3 and D7, but not 5 µM. Compared to the controls, ZrO(NO_3_)_2_ at 50 and 500 µM up-regulated BSP expression at D7, but not at D3; whereas ZrCl_4_ at 50 µM and 500 µM increased BSP expression at D3 and D7 (Fig. 3B). Runx2 gene expression was significantly increased by ZrO(NO_3_)_2_ and ZrCl_4_ at 5 and 50 µM at D3 (Fig. 3C). At D7, ZrO(NO_3_)_2_ at all three different concentrations increased Runx2 expression. However, ZrCl_4_ increased Runx2 expression only at 50 and 500 µM. OC was evaluated by real time PCR as a gene marker for later osteogenesis. ZrO(NO_3_)_2_ and ZrCl_4_ at 500 µM .increased OC expression at D7 but not at D3. At D3, OC expression was up-regulated by ZrCl_4_ at 50 µM; however it was suppressed by ZrO(NO_3_)_2_ at 5 and 500 µM and ZrCl_4_ at 5 µM (Fig. 3D). ALP expression was up-regulated by ZrCl_4_ at 50 µM at D3 but no consistent evidence of regulation of ALP gene expression was seen (Fig. 3E).

Overall, ZrO(NO_3_)_2_ and ZrCl_4_ strongly increased osteogenic gene expression in the HOBs cultured for 3 and/or 7 days. The upregulation of osteogenic genes indicates that Zr induces osteogenesis in primary HOBs.

To further investigate the mechanism of Zr-induced early osteogenesis, BMP2 genes and BMP receptor genes were analyzed in HOBs cultured under different treatments with Zr solutions and control media.

**Figure 4:**
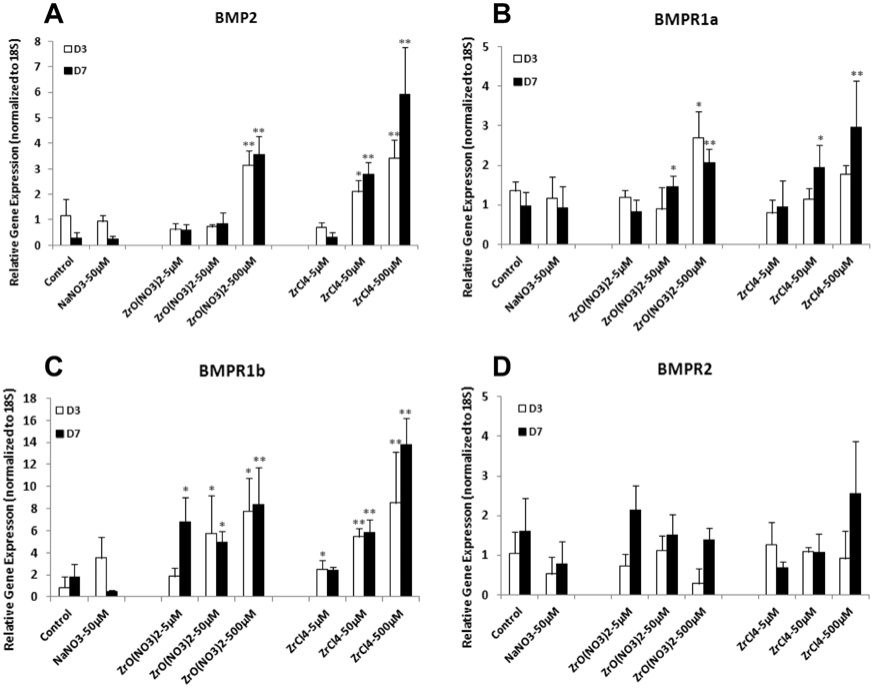
Zirconium ions up-regulate gene expression of BMP2 and BMPR in HOBs. HOBs were treated with ZrO(NO_3_)_2_ and ZrCl_4_ solutions at the concentrations of 5, 50 and 500 µM for 3 and 7 days. Real-time PCR was employed to analyze the gene expression of BMP2 (A), BMP receptors BMPR1a (B), BMPR1b (C) and BMPR2 (D). (A) At D3 and D7, BMP2 gene expression was significantly up-regulated in HOBs treated with ZrO(NO_3_)_2_ at 500 µM and ZrCl_4_ at 50 µM and 500 µM, compared to the control. (B) At D3, BMPR1a expression was up-regulated in HOBs by ZrO(NO_3_)_2_ and ZrCl_4_ only at 500 µM, compared to the control. ZrO(NO_3_)_2_ and ZrCl_4_ at 50 and 500 µM significantly increased BMPR1a expression in HOBs treated for 7 days. BMPR1a gene expression was inhibited by ZrCl_4_ at 5 µM at D3. (C) BMPR1b expression was significantly increased in HOBs treated with ZrO(NO_3_)_2_ and ZrCl_4_ at 50 and 500 µM at D3 and D7. Increased BMPR1b expression was exhibited by ZrO(NO_3_)_2_ at 5 µM only at D7 whereas ZrCl_4_ at 5 µM only at D3, respectively, compared with the control. (D) Zirconium solutions had no effect on the BMPR2 expression in HOBs.*p<0.05, **p<0.01 vs control.

At D3 and D7, BMP2 gene expression was significantly up-regulated in HOBs treated with ZrO(NO_3_)_2_ at 500 µM and ZrCl_4_ at 50 µM and 500 µM, compared to the control (Fig. 4A). For BMPR gene expression at D3, BMPR1a expression was up-regulated compared to the control in HOBs by ZrO(NO_3_)_2_ and ZrCl_4_ only at 500 µM. In HOBs treated for 7 days ZrO(NO_3_)_2_ and ZrCl_4_ at 50 and 500 µM significantly increased BMPR1a expression (Fig. 4B). BMPR1a gene expression seemed being inhibited by ZrCl_4_ at 5 µM at D3. BMPR1b expression was significantly increased in HOBs treated with ZrO(NO_3_)_2_ and ZrCl_4_ at 50 and 500 µM at D3 and D7. Increased BMPR1b expression was exhibited by ZrO(NO_3_)_2_ at 5 µM only at D7 whereas ZrCl_4_ at 5 µM only at D3, respectively, compared with the control (Fig. 4C). Zirconium solutions had no effect on the BMPR2 expression in HOBs (Fig. 4D).

Generally, higher concentrations of ZrO(NO_3_)_2_ and ZrCl_4_ (500 µM) significantly increased the expression of BMP2 and BMP receptor genes BMPR1a and BMPR1b at D3 and D7 (Fig. 4A-C).

### Zr ions activate the BMP/SMAD1/5 signaling pathway

BMP/SMAD signaling pathways play critical roles in the regulation of osteoblast differentiation. BMPs exert their functions through binding to Ser/Thr kinase receptors in the membrane and subsequently phosphorylating the downstream effectors SMAD1 and SMAD5 at the Ser463 and S465 sites. Phosphorylated SMAD1/5 is then translocated into the nucleus to activate the target gene transcription.

**Figure 5:**
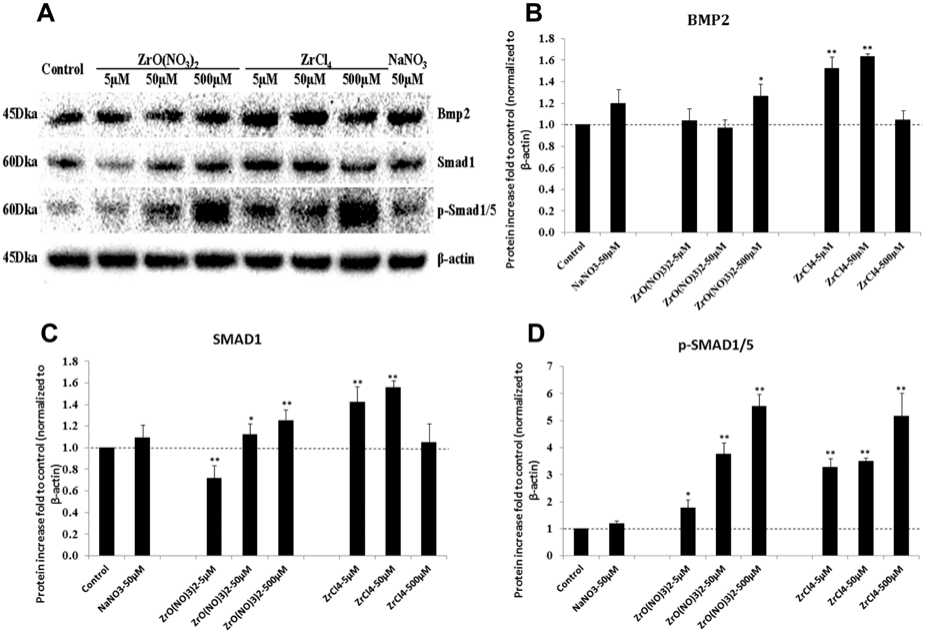
Zirconium ions elevate protein levels in the BMP/SMAD signaling pathway. (A) Western blotting was used to determine the expression levels of BMP2, SMAD1 and phospho-SMAD1/5 (p-SMAD1/5) in primary HOBs treated with ZrO(NO3)2 and ZrCl_4_ for 7 days. β-actin was used as the loading control. (B) Quantification of Bmp2 protein bands indicated ZrO(NO3)2 at 500 µM increased Bmp2 protein level compared to the untreated and NaNO_3_ controls. Compared to the controls, ZrCl_4_ treatment increased BMP2 at the concentrations of 5 and 50 µM, but not at 500 µM. (C) Treatment of HOBs with ZrO(NO_3_)_2_ at 50 and 500 µM increased SMAD1 protein expression, but SMAD1 levels were reduced at 5 µM, compared to the controls. ZrCl4 treatment increased SMAD1 protein level at 5 and 50 µM but not 500 µM. (D) Both ZrO(NO_3_)_2_ and ZrCl_4_ treatment significantly and dose dependently increased the p-SMAD1/5 levels in HOBs. *p<0.05, **p<0.01 vs control.

As the BMP2 and its receptor gene expression was up-regulated by Zr chemicals, we further investigated the expression of BMP2 protein levels and its downstream effectors of SMAD1 and p-SMAD1/5. Western blotting was employed to evaluate the protein levels of BMP2, SMAD1 and p-SMAD1/5 in HOBs cultured with different Zr solutions and control media for 7 days (Fig. 5A). After normalizing the protein levels to those of β-actin, BMP2 protein found to be increased 20% in HOBs after treatment with ZrO(NO_3_)_2_ at 500 µM and from 40% to 60% with ZrCl4 treatment at 5 and 50 µM (Fig. 5B). The results showed that Zr ions significantly activated the protein levels of SMAD1 as well as p-SMAD1/5 in HOBs (Fig. 5C, D). Furthermore, the dose-response effects with steady increase of p-SMAD1/5 protein in Zr-treated HOBs cultured with low to high concentrations is consistent with the activation of SMAD1/5 signaling pathway.

**Figure 6:**
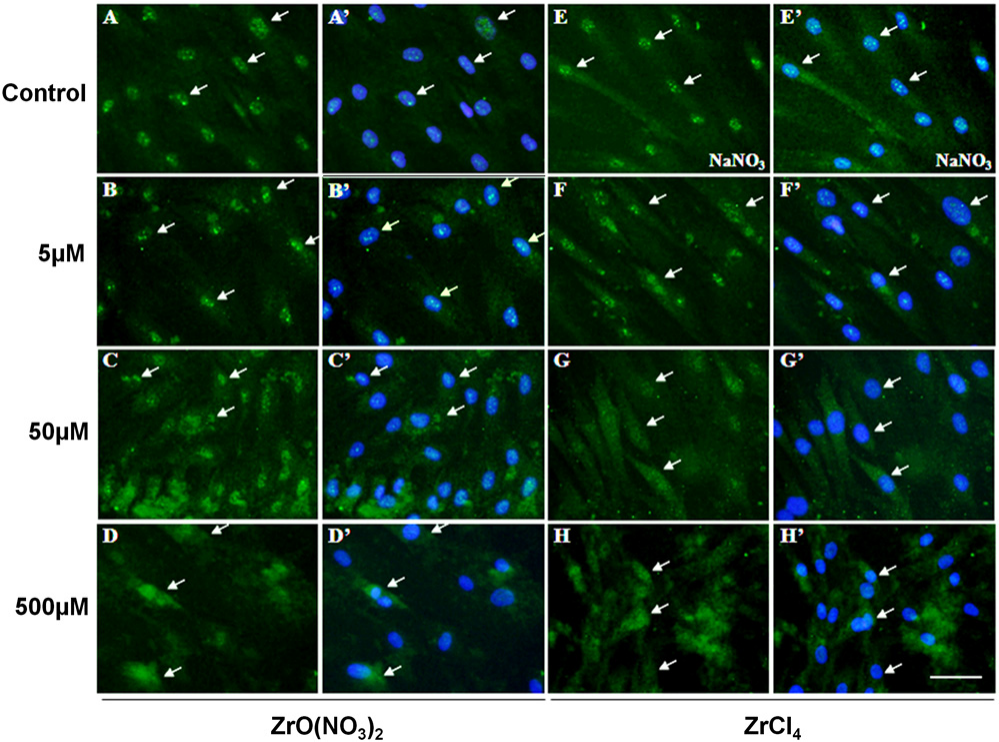
Localization of BMP2 in HOBs treated with zirconium solutions. In untreated control (A, A’) and NaNO_3_-reated control (E,E’)HOBs, BMP2 localized strongly in the cell nuclei (arrows) but weakly detected in the cytoplasm. In ZrO(NO_3_)_2_-treated HOBs (B-D), BMP2 is strongly localized in nuclei. Localization of BMP2 protein is also strongly observed in the cytoplasm of HOBs treated with ZrO(NO_3_)_2_ at 50 and 500 µM (arrows, C,C’,D,D’). Localization of BMP2 in ZrCl_4_-treated HOBs (F-H) is similar to that in ZrO(NO_3_)_2_-treated HOBs. Scale bar: 50 µm.

**Figure 7:**
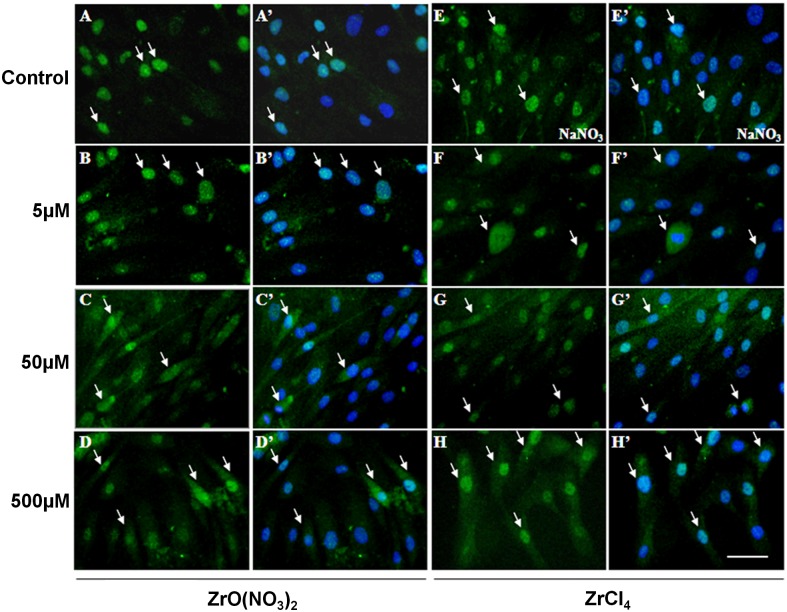
Localization of phospho-SMAD1/5 in HOBs treated with zirconium solutions. Strong p-SMAD1/5 nucleus localization is detected in the control (arrows, A,A’) and NaNO_3_-reated control (E,E’) HOBs. In HOBs treated with ZrO(NO_3_)_2_ at 5 µM (B,B’), p-SMAD1/5 has similar localization in the nucleus as in controls. (C-D) In the HOBs treated with ZrO(NO3)2 at 50 and 500 µM, p-SMAD1/5 localization is strongly detected the nuclei and in cytoplasm (arrows, C,C’,D’D’). Treated with ZrCl4 at different concentrations of 5, 50 and 500 µM, HOBs show the similar p–SMAD1/5 localization exhibited in the nucleus and/or cytoplasm, as seen in HOBs treated with ZrO(NO_3_)_2_. Scale bar: 50 µm.

To confirm the activation of BMP2 and p-SMAD1/5 protein in HOBs by Zr ions, immuno-staining was carried out to detect the protein localization in cells. HOBs were treated with Zr solutions and control media at different concentrations for 7 days, and then were incubated with anti-BMP2 or anti-p-SMAD1/5 antibodies for immunofluorescence. BMP2 was localized strongly in the HOB nuclei and weakly in the cytoplasm in untreated control (arrows, Fig. 6A,A’) and NaNO_3_ control (Fig. 6E,E’). In Zr-treated HOBs, BMP2 reactivity was significantly elevated (Fig. 6B-D, F-H) compared with the controls. BMP2 accumulation was strongly observed in the nuclei and cytoplasm in the Zr treatment groups at 50 and 500 µM (Fig. 6C,D,G,H). p-SMAD1/5 was localized in the HOB nuclei and not cytoplasm in controls (arrows, Fig. 7A, E). However, in HOBs treated with Zr solutions, p-SMAD1/5 was observed to appear strongly in the nuclei and weakly in the cytoplasm (Fig. 7C,D,G,H). These results indicate that Zr ions significantly stimulate BMP2 protein expression and subsequently stimulate SMAD1/5 phosphorylation and pathway activation.

### Noggin suppressed osteogenic gene expression in HOBs co-incubated with ZrCl_4_

**Figure 8:**
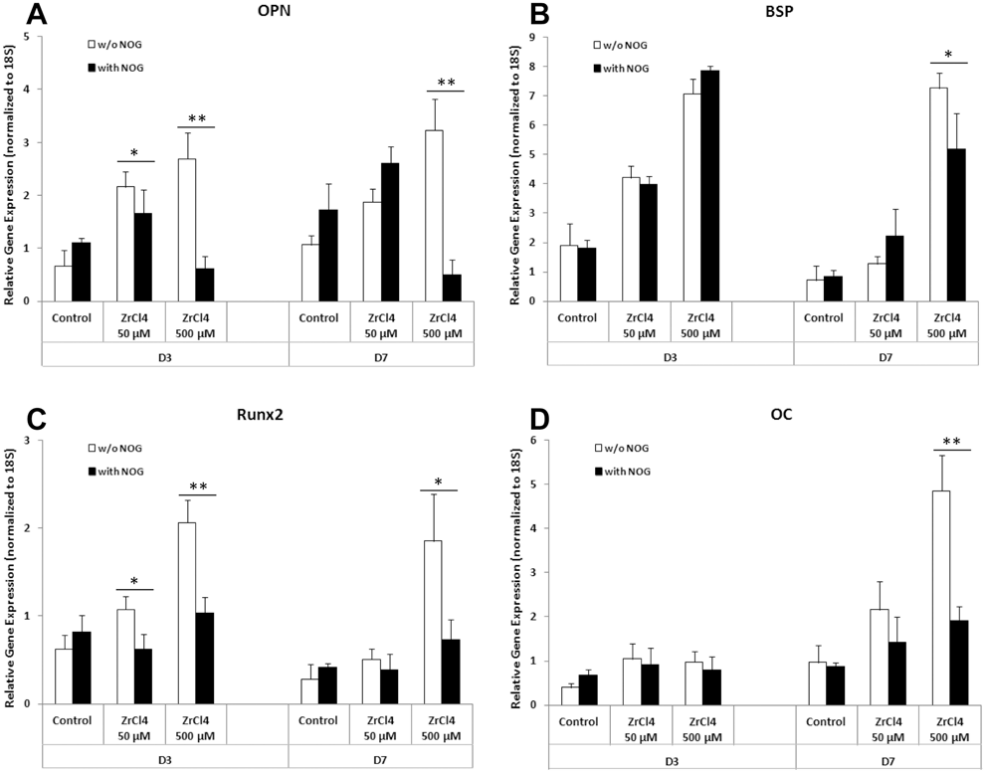
Noggin suppressed osteogenic gene expression in HOBs treated with ZrCl_4_. Combined culture of HOBs with ZrCl_4_ and noggin (500 ng/ml) suppressed: (A) OPN gene expression at D3 (ZrCl_4_ at 50 and 500 µM) and D7 (ZrCl_4_ at 500 µM); (B) BSP gene expression at D7 (ZrCl_4_ at 500 µM); (C) Runx2 gene expression at D3 (ZrCl_4_ at 50 and 500 µM) and at D7 D7 (ZrCl_4_ at 500 µM); and (D) OC gene expression at D7 (ZrCl_4_ at 500 µM). *p<0.05, **p<0.01 vs the same concentration of ZrCl_4_.

In this study we have shown that Zr ion up-regulated the gene expression of BMP2 and BMP receptor genes as well as promoted the proteins levels of BMP2 and the downstream effectors SMAD1 and pSMAD1/5 in BMP signaling pathway. To investigate whether the osteogenic effects of Zr ions were mediated by activation of the BMP signaling pathway, Noggin, an inhibitor of BMP signaling pathway, was added to HOB cultures in the presence of ZrCl_4_ at the concentrations of 50 and 500 µM for 3 and 7 days. Real time PCR results showed that the osteogenic genes OPN, BSP, Runx2 and OC, were upregulated in HOBs by ZrCl_4_ at concentrations of 50 and 500 µM at D3 and D7 (Fig. 8). These results were consistent with the results shown above (Fig. 3). Addition of Noggin to these cultures significantly suppressed the increase in gene expression of OPN, BSP, Runx2 and OC at day 7 at a 500 µM ZrCl_4_ concentration. Noggin also significantly reduced expression of RUNX2 and OPN at D3 at this concentration. These results provide additional evidence the the effects of Zr ions on osteogenesic differentiation of HOBs is dependent on BMP2 signaling.

## Discussion

This study identifies that Zr ions have a novel osteogenic activity on primary human osteoblasts. Zr ions were found to both increase the proliferation of human osteoblast precursors and to enhance their differentiation into osteoblasts. These HOBs were derived from discarded human bone fragments removed at surgery to correct scoliosis and come from young healthy individuals without systemic illnesses. The primary cultures of these cells initially represent a population of committed osteoblast precursors Early in culture they actively proliferate but later proliferation slows and the processes of differentiation predominate [25–29]. In the present study, we have identified the ability of Zr ions to increase the proliferation of HOBs at early stages of culture. Growth in cell number was associated with increased cell expression of the cell proliferation marker Ki67 indicating increased cell mitosis, rather than reduction in apoptosis, mediated the increase in viable cell number.

Later in culture (day 21), and under minimal osteogenic conditions (culture with ascorbic acid), HOBs have the ability to mature into osteoblasts and are then able to lay down collagen matrix and stimulate its mineralization. During this differentiation process, various changes in gene expression occur under the influence of autocrine signaling mediated by growth factors such as BMP’s and Wnt’s. We have demonstrated that Zr ions are able to promote the later differentiation of HOBs and enable them to lay down and mineralize bone matrix. Treatment with Zr ions increased the amount of mineralized matrix deposited after 21 and 28 days of HOB culture under osteogenic conditions. This increase in mineralization was preceded at early stages of culture by increased expression of genes characteristic of osteoblasts. OPN and BSP were increased at day three. Osteocalcin (OC), which is a later marker of osteoblast differentiation, was down–regulated at day 3 but up-regulated later at day 7 of culture. The initial down regulation of OC is consistent with an initial proliferative action of Zr that delayed differentiation, followed by an enhancement of differentiation later in the culture period.

We have previously shown that growth of primary human osteoblasts on Zr containing ceramic scaffolds causes as induction of BMP2 expression [8]. We investigated in this study whether a similar response to soluble Zr ions could be detected. We found that both forms of Zr used were able to increase the gene expression of BMP2 by HOBs and in addition increases in BMPR1a and BMPR1b were also seen indicating that Zr ions may sensitize osteoblasts to BMP-mediated effects. Consistent with an activation of BMP2 signaling was the marked increase in phosphorylated SMAD1/5 levels in both the cytoplasm and nucleus of HOBs treated with Zr ions. The increase in total levels of phosphorylated SMADs observed and the increase in their nuclear localization indicate the activation of these transcription factors through BMP ligand interaction with BMP receptors and their potential ability to control osteoblastic gene expression in response to Zr ions. BMP2 is a potent stimulator of osteoblast differentiation [30] and its increased expression and the marked increase in phosphorylated SMAD1/5 provide convincing evidence that BMP2 is a mediator of the osteogenic effects of Zr ions. Suppression of osteogenic gene expression by noggin, an inhibitor of the BMP signaling pathway, in HOBs co-treated with Zr ions provides additional evidence that osteogenesis induced by Zr ions is associated with BMP signaling. The molecular interaction of Zr ions with either BMP receptors or upstream regulators remains unidentified and we have not investigated Zr ion effects on other pathways important in osteogensis such as wnt and parathyroid hormone receptor signaling which may also be modulated by Zr. These questions remain the subject of ongoing research. The effects of Zr were most pronounced at the concentrations of 50 µM and 500 µM. The relevance of the higher of these concentrations to in vivo findings is currently uncertain as simple dissolution studies of Zr containing calcium silicate ceramics in simulated body fluid show relatively low levels of release [6]. Clearly local levels of Zr adjacent to degrading materials would be much higher than those reported in that study, but the relevance of our findings to what can be achieved in the local bone environment remains to be confirmed.

Two sources of Zr ions were used, ZrO(NO_3_)_2_ and ZrCl_4_. ZrO(NO_3_)_2_ is the form we used in preparation of Zr containing ceramics by the sol-gel method [7], and ZrCl_4_ represents the simplest available soluble source of Zr ions. Both of these forms had similar overall effects on the various parameters assessed. The addition of NO_3_ ions had no effect on HOBs indicating that the effects seen with ZrO(NO_3_)_2_ treatment was indeed indicative of a Zr effect. We have not assessed non-soluble forms of Zr in the current study and it is possible that Zr containing particulate debris could have different actions including inflammatory effects as observed for other prosthesis materials.

## Conclusion

We have identified a novel activity of Zr ions on the differentiation of human osteoblasts. This finding is of interest given the potential benefits of improving the osteogenic properties of orthopaedic implants with a process as simple as incorporating labile Zr-containing materials into orthopaedic implants from where it could be released to enhance bone ingrowth with resultant improvement of implant stability and longevity.
